# Influence of preactivation on fascicle behavior during eccentric contraction

**DOI:** 10.1186/s40064-016-2550-5

**Published:** 2016-06-17

**Authors:** Atsuki Fukutani, Jun Misaki, Tadao Isaka

**Affiliations:** Research Organization of Science and Technology, Ritsumeikan University, 1-1-1 Noji-higashi, Kusatsu, Shiga 525-8577 Japan; Japan Society for the Promotion of Science, 5-3-1 Kojimachi, Chiyoda-ku, Tokyo, 102-8472 Japan; Graduate School of Sport and Health Science, Ritsumeikan University, 1-1-1 Noji-higashi, Kusatsu, Shiga 525-8577 Japan; Faculty of Sport and Health Science, Ritsumeikan University, 1-1-1 Noji-higashi, Kusatsu, Shiga 525-8577 Japan

**Keywords:** Ultrasonography, Vastus lateralis, Knee extension, Force length, Force velocity, Muscle–tendon complex

## Abstract

**Background:**

Because muscle fascicle behavior affects to the force-generating capability, understanding of muscle fascicle length changes during dynamic movements is important. Preactivation may affect the muscle fascicle length changes, especially in the case of eccentric contraction. However, its influence has not been clarified. To this end, muscle fascicle behavior during eccentric contraction was compared between preactivation and no-preactivation conditions.

**Results:**

Seven healthy men (24.6 ± 2.2 years, 169 ± 2 cm, 68.0 ± 5.1 kg) participated in this study. An eccentric knee extension controlled by a Biodex system was adopted as the testing motion. Muscle fascicle behavior of vastus lateralis during eccentric knee extensions was compared following two conditions. In preactivation condition, isometric preactivation was conducted before initiating eccentric contraction. On the other hand, in no-preactivation condition, muscle contraction was initiated immediately after initiating the knee angle change induced by a dynamometer. The muscle fascicle length at the onset of eccentric contraction was significantly shorter in preactivation condition than in no-preactivation condition (Cohen’s *d* = 0.98, *p* < 0.001) although that at the end of eccentric contraction was not different (Cohen’s *d* = 0.08, *p* = 0.844). The muscle fascicle was elongated throughout the eccentric contraction phase in preactivation condition. On the other hand, muscle fascicle was shortened in the first part, and then elongated in the latter part of the eccentric contraction phase in no-preactivation condition.

**Conclusions:**

The muscle fascicle is shortened even during eccentric contraction phase. However, this shortening is disappeared when preactivation is conducted.

## Background

Because changes in muscle fascicle length affect the force-generating capability based on force–length (Gordon et al. 1966) and force–velocity (Hill 1938) relationships, clarifying the precise behavior of the muscle fascicle is important for better understanding human locomotion.

Previous studies (Fukunaga et al. 2001; Ishikawa et al. 2007) reported that, in the eccentric contraction phases (“eccentric contraction” refers to the elongation of muscle–tendon complex based on joint angle changes not muscle fascicle length changes), the muscle fascicle behaved isometrically during the eccentric contraction phase. On the other hand, Chino et al. (2009) and Wakahara et al. (2009) reported that muscle fascicle was elongated during the eccentric contraction phase. These results indicate that muscle fascicle behavior during eccentric contraction phase differs depending on circumstances. One of the possible reason would be preactivation. In this context, preactivation means prior activation before the eccentric contraction phase. Fukunaga et al. (2001) and Ishikawa et al. (2007) did not adopt substantial preactivation. Specifically, muscle contraction was initiated during the muscle–tendon complex was elongated. On the other hand, Chino et al. (2009) and Wakahara et al. (2009) conducted preactivation. Specifically, isometric contraction was conducted before the muscle–tendon complex was elongated. This difference would affect to the muscle fascicle behavior during eccentric contraction phase. Therefore, the purpose of this study was to examine the influence of preactivation on muscle fascicle behavior during a subsequent eccentric contraction phase.

## Methods

### Subjects

Seven healthy men (mean ± SD, 24.6 ± 2.2 years, 169 ± 2 cm, 68.0 ± 5.1 kg) were recruited into this study. The purpose and risks were explained to each participant and written informed consent was obtained from all participants. The Ethics Committee on Human Research of the Ritsumeikan University approved this study (BKC-IRB-2014-026).

### Experimental procedures and measurements

Knee extensors of right thigh were adopted as target muscles. Two trials were conducted. The first trial consisted of eccentric contraction without preactivation (no-preactivation condition). The second trial involved eccentric contraction with isometric preactivation (preactivation condition). Participants sat on a dynamometer (Biodex, SAKAImed, Japan) with the hip joint flexed 80° and with the knee joint flexed 40° (the hip and knee joint angles at the anatomical position were defined as 0°, respectively). The other joint angles were not strictly controlled because primary knee extensors (quadriceps femoris) are involved only in knee and hip joints. Following instruction was given to the participants, “Keep the same posture throughout the experiment”. Range of motion and lengthening velocity of the eccentric contraction were identical between the preactivation and no-preactivation conditions (range of motion: from 40° to 100° flexion, lengthening velocity: 90°/s). To obtain maximal intensity contraction, following verbal instruction, “as strong as possible”, was given to the participants. The onset of muscle contraction in the no-preactivation condition was immediately after the commencement of dynamometer’s movement. By contrast, in the preactivation condition, the onset of muscle contraction was approximately 1.5 s before the commencement of dynamometer’s movement. Considering our preliminary experiment, this duration was long enough to obtain maximal isometric torque in the preactivation phase. Trials were separated by a rest time of more than 2 min. Before experiments, the sequence of conditions was determined randomly. As a result, five out of seven conducted preactivation condition first, while two out of seven conducted no-preactivation condition first.

The muscle fascicle length of the vastus lateralis was measured by using ultrasonography (SSD-3500, ALOKA, Japan). Location of the ultrasonographic probe was near the middle of the vastus lateralis. Because of the limited field of view (6 × 6 cm) of the ultrasonographic probe (UST-5710, ALOKA, Tokyo, Japan), a linear extrapolation method (Ema et al. 2013; Power et al. 2013) was adopted. Once the best position and orientation of the ultrasonographic probe were found, ultrasonograohic probe was tightly fixed by underwrap and surgical tape. Sampling frequency of ultrasonographic images was 30 Hz. Muscle fascicle length and pennation angle at the onset of eccentric contraction and at the end of eccentric contraction were calculated. These values were compared between the preactivation and no-preactivation conditions.

### Statistics

Descriptive data are presented as mean ± SD. Two-way analysis of variance (ANOVA) with repeated measures was conducted (with/without preactivation × onset/end of the eccentric contraction) for muscle fascicle length and pennation angle, respectively. If the interaction was significant, paired *t* test was applied to compare muscle fascicle length and pennation angle at the onset and end of the eccentric contraction between the preactivation and no-preactivation conditions. Effect size was shown as the partial *η*^2^ and Cohen’s *d* for ANOVA and paired *t* test, respectively. The level of statistical significance was set at *p* < 0.05. Statistical analyses were performed by SPSS 20.0 (IBM, Japan).

## Results

**Fig. 1:**
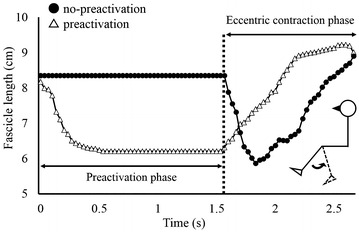
Typical example of the muscle fascicle length changes in the preactivation and no-preactivation conditions (N = 1)

Typical example of muscle fascicle behavior is shown in Fig. 1. Generally, the muscle fascicle was elongated throughout the eccentric contraction phase in the preactivation condition, while the muscle fascicle was shortened in the first part and then elongated in the latter part of the eccentric contraction phase in the no-preactivation condition. These muscle fascicle behavior were observed from all subjects.

For muscle fascicle length, two-way ANOVA with repeated measures revealed that significant interaction was found (partial *η*^2^ = 0.970, *p* < 0.001). Subsequent analyses demonstrated that muscle fascicle length significantly differed between the preactivation and no-preactivation conditions at the onset of eccentric contraction (Cohen’s *d* = 0.98, *p* < 0.001), but not at the end of eccentric contraction (Cohen’s *d* = 0.08, *p* = 0.844; Table 1, upper panel).

**Table 1:** Muscle fascicle lengths and pennation angles at the onset and end of eccentric contraction

	Mean ± SD (cm)	*p* value	Effect size (Cohen’s *d*)
*Muscle fascicle length*			
Onset of ECC			
Preactivation	6.6 ± 0.7	<0.001	0.98
No-preactivation	9.2 ± 1.1		
End of ECC			
Preactivation	9.0 ± 1.1	0.844	0.08
No-preactivation	8.9 ± 1.5		

	Mean ± SD (°)	*p* value	Effect size (Cohen’s *d*)
*Pennation angle*			
Onset of ECC			
Preactivation	21.1 ± 3.2	0.004	0.90
No-preactivation	18.4 ± 2.8		
End of ECC			
Preactivation	16.9 ± 2.7	0.169	0.21
No-preactivation	16.4 ± 2.4		

As is the case with muscle fascicle length, significant interaction was found (partial *η*^2^ = 0.765, *p* = 0.004), and significant difference was found between the preactivation and no-preactivation conditions at the onset of eccentric contraction (Cohen’s *d* = 0.90, *p* = 0.004), but not at the end of eccentric contraction (Cohen’s *d* = 0.21, *p* = 0.169; Table 1, lower panel).

## Discussion

The purpose of this study was to examine the influence of preactivation on muscle fascicle behavior during a subsequent eccentric contraction phase. Muscle fascicle behavior clearly differed between the preactivation and no-preactivation conditions. In the preactivation condition (Fig. 1, white triangles), the muscle fascicle shortened when isometric preactivation was initiated, and then, its length remained the same during the isometric preactivation phase. Once the joint angle began to change (i.e., muscle–tendon complex began to elongate) the muscle fascicle was also elongated. Consequently, joint movement and muscle fascicle behavior were similar during the eccentric contraction phase. By contrast, in the no-preactivation condition (Fig. 1, black circles), fascicle length was shortened during the first phase in association with the initiation of muscle contraction although muscle–tendon complex was elongated. Changes in pennation angle was similar with those in muscle fascicle length.

Our data indicate that the discrepancy of muscle fascicle behavior during the eccentric contraction phase across previous studies can be explained by whether preactivation was conducted. In studies reporting elongation during eccentric contraction phase (Chino et al. 2009; Wakahara et al. 2009) muscle contraction was initiated before the muscle–tendon complex was elongated. By contrast, in studies reporting shortening or isometric behavior during eccentric contraction phase (Fukunaga et al. 2001; Ishikawa et al. 2007; Hirayama et al. 2012), muscle contraction was initiated while the muscle–tendon complex was elongated. Thus, fascicle shortening occurred in the initial phase of the muscle contraction should lead to the distinct behavior of the muscle fascicle during the eccentric contraction phase. The likely reason for this different fascicle behavior would be tendon elongation (i.e., muscle tendon interaction; Sano et al. 2015; Farris et al. 2016) and/or eliminating slack (Herbert et al. 2015; Hirata et al. 2016) at the onset of contractions, which modulate the muscle fascicle length at a given joint angle.

In no-preactivation condition, it took 236 ± 44 ms (mean ± SD) to complete muscle fascicle shortening during the initial phase of eccentric contraction phase. Thus, when the contraction duration is relatively short such as less than 200 ms, the muscle fascicle would continue shortening even during eccentric contraction phase. Taken this into account, muscle fascicle length changes, not joint angle changes, should be observed, especially, when the movement duration is relatively short and preactivation is not conducted. For example, when contraction velocity is fast and/or range of motion is small, contraction duration becomes short. Muscle fascicle behavior during these contractions, which may be frequently observed in sport activities, would be strongly affected by preactivation.

Although our sample size was small (N = 7), the results of different muscle fascicle behavior between preactivation and no-preactivation conditions should be robust. This is because muscle fascicle shortening at the onset of eccentric contraction in no-preactivation condition was confirmed from all participants (Fig. 1, black circles), and muscle fascicle did not shorten at all during eccentric contraction phases in the preactivation condition (Fig. 1, white triangles).

## Conclusions

Muscle fascicle behavior during eccentric contraction (judging from joint angle changes) was clearly different between preactivation and no-preactivation conditions. These results indicate that when preactivation is not conducted, muscle fascicle behavior during eccentric contraction phase does not necessarily correspond with joint angle changes. Thus fascicle length changes not joint angle changes should be considered, especially in the early phase of eccentric contractions.
